# Mechanism of internalization of MDA-7/IL-24 protein and its cognate receptors following ligand-receptor docking

**DOI:** 10.18632/oncotarget.27150

**Published:** 2019-08-20

**Authors:** Anjan K. Pradhan, Praveen Bhoopathi, Sarmistha Talukdar, Swadesh K. Das, Luni Emdad, Devanand Sarkar, Andrei I. Ivanov, Paul B. Fisher

**Affiliations:** ^1^ Department of Human and Molecular Genetics, Virginia Commonwealth University, School of Medicine, Richmond, VA, USA; ^2^ VCU Institute of Molecular Medicine, Virginia Commonwealth University, School of Medicine, Richmond, VA, USA; ^3^ VCU Massey Cancer Center, Virginia Commonwealth University, School of Medicine, Richmond, VA, USA; ^4^ Department of Inflammation and Immunity, Lerner Research Institute at Cleveland Clinic, Cleveland, OH, USA

**Keywords:** MDA-7/Interleukin-24, Interleukin receptor, recombinant protein

## Abstract

Melanoma differentiation associated gene-7 (*mda-7/IL-24*) is a member of the IL-10 family of cytokines, with ubiquitous direct and “bystander” tumor-selective killing properties. MDA-7/IL-24 protein binds distinct type II cytokine heterodimeric receptor complexes, IL-20R1/IL-20R2, IL-22R1/IL-20R1 and IL-22R1/IL-20R2. Recombinant MDA-7/IL-24 protein induces endogenous *mda-7/IL-24* expression in a receptor-dependent manner; since A549 cells that lack a complete set of cognate receptors are not responsive to exogenous protein. The mechanism of MDA-7/IL-24 ligand-receptor biology is not well understood. We explored the interaction of MDA-7/IL-24 with its’ receptors and the consequences of ligand-receptor docking. Using both pharmacological and genetic approaches we demonstrate that MDA-7/IL-24 internalization employs the clathrin-mediated endocytic pathway leading to degradation of receptors via the lysosomal/ubiquitin proteosomal pathway. This clathrin-mediated endocytosis is dynamin-dependent. This study resolves a novel mechanism of MDA-7/IL-24 protein “bystander” function, which involves receptor/protein-mediated internalization and receptor degradation.

## INTRODUCTION

The IL-10 gene family member *mda-7 (melanoma differentiation associated gene-7)* also known as Interleukin-24 (IL-24) is a well-characterized multifunctional tumor suppressor displaying broad-spectrum cancer-specific cell killing activity [1–6]. *mda-7/IL-24* was first identified and cloned using a differentiation induction subtraction hybridization (DISH) screening approach with metastatic human melanoma cells induced to terminally differentiate by treatment with recombinant human interferon and the protein kinase C activator mezerein [7, 8]. *mda-7/IL-24* displays restricted expression, which is evident in melanocytes, peripheral blood leukocytes and a subset of immune cells [5, 6, 9]. MDA-7/IL-24 protein expression is low or absent in cancer cells as compared to their normal counterparts [5]. The near ubiquitous anti-tumor functions and the underlying mechanisms of cancer-selective induction of apoptosis and toxic autophagy have been extensively investigated in melanoma and many additional cancers [4–7, 10, 11]. Like other cytokines of the IL-10 family, MDA-7/IL-24 is a secreted protein [5, 12]. MDA-7/IL-24 is an evolutionarily conserved protein that regulates a diverse array of signaling pathways and alters the expression of multiple apoptotic molecules in cancer cells including Bcl-2 [13], Bcl-xL [13], NOXA [14], and AIF [15]. MDA-7/IL-24 induces the expression of the chaperone protein BiP/GRP78, which in turn results in endoplasmic reticulum (ER) stress and cell death [16]. Additionally, this cytokine regulates toxic autophagy through a miR-221-Beclin-1 axis [17, 18]. MDA-7/IL-24 also has anti-angiogenic activity and it inhibits invasion and metastasis of cancer cells (1). Moreover, following receptor engagement, MDA-7/IL-24 can stimulate its own production through a paracrine/autocrine loop [19]. As a secreted cytokine, MDA-7/IL-24 induces growth regression in distant tumors, hence exerts a potent “bystander activity” [20].

Previous studies have shown that MDA-7/IL-24 induces apoptosis only in cancer cells that have a complete set of functional IL-20R/IL-22R receptor pairs, but cancer cells that lack a complete set of receptors can escape the MDA-7/IL-24 mediated cell death [12, 21]. Exogenously applied MDA-7/IL-24 protein does not affect A549 cells that lack a pair of cognate MDA-7/IL-24 receptors, IL-20R2 and IL-22R1, indicating that this response is mediated by binding of MDA-7/IL-24 to its cognate receptors [12]. But the exact mechanism defining ligand-receptor biology of MDA-7/IL-24 is not well deciphered. Since MDA-7/IL-24 is a recombinant protein and signals through a receptor dependent manner, there is a need to study the mechanism of MDA-7/IL-24-mediated signaling and the fate of the receptors.

Historically, analyzing endocytic pathways have proven to be the most robust approach to scrutinize ligand-induced cellular signaling [22]. Receptor-mediated endocytosis is one of the major physiological processes maintaining cellular homeostasis and several ligands with their receptors utilize this pathway [23]. Receptor-independent endocytosis can also occur in specific contexts [23]. Receptor-dependent endocytosis can either be clathrin-dependent or caveolae-dependent [23]. Several conventional mechanisms regulating ligand recruitment to clathrin coated pits have been described [24]. Clathrin-mediated endocytosis is a well-established method for the internalization of a diverse array of cargo proteins [24]. This leads to endosomal processing of both the ligand and the receptor [24], and then the receptors are either recycled to the surface or undergo degradation mediated by proteasomes or lysosomes [25]. A complex network involving a number of molecules forms the invaginated clathrin pits and endocytic vesicles [25]. Several accessory molecules including actin and dynamins play a key role in addition to clathrin in this process [26]. Dynamin activity is necessary for generation of endocytic carriers both for clathrin- and caveolae-mediated endocytosis. There are also dynamin-independent endocytic pathways for various surface- and lipid-bound proteins, such as MHC-I (major histocompatibility complex) [27]. In this study, we explored the interaction of MDA-7/IL-24 with its cognate receptors and explored the fate of both the ligand and the receptor following ligand-receptor engagement. Here we show that upon docking to receptors, MDA-7/IL-24 is internalized via endocytosis and this process is clathrin-dependent. We also uncover a role of the lysosomal/ubiquitin proteosomal pathway, and Dynamin in this dynamic physiological process.

## RESULTS

### Ligand-dependent MDA-7/IL-24 receptor internalization

**Figure 1:**
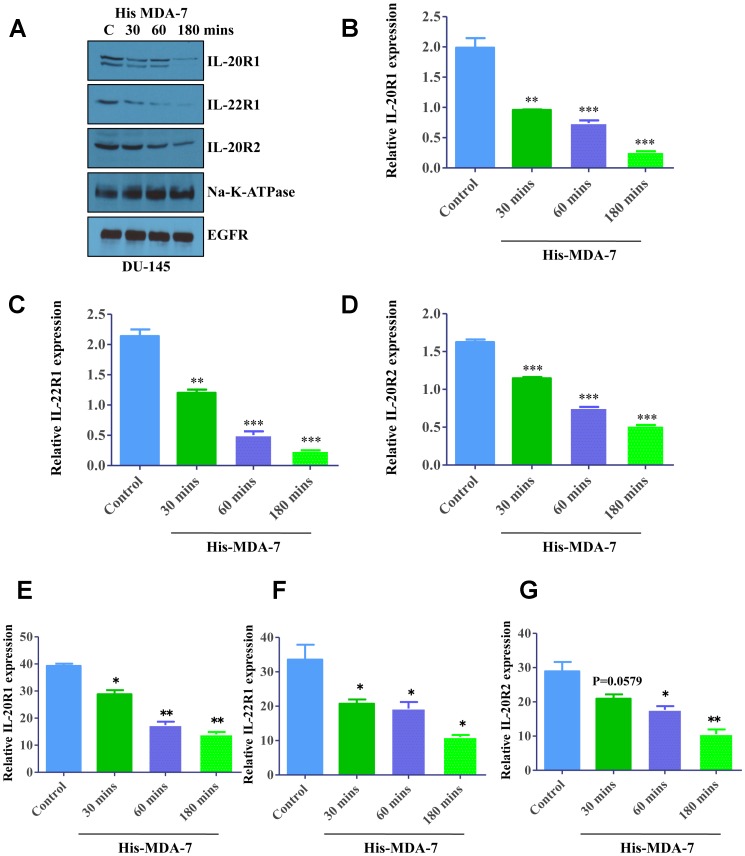
Ligand-induced internalization of MDA-7/IL-24 receptors. (**A**) Western blotting showing the expression of receptors in a time-dependent manner following treatment with His-MDA-7/IL-24 protein. DU-145 cells were treated with His-tagged MDA-7/IL-24 protein for the times indicated; biotinylated membrane fractions were isolated and probed with the indicated antibodies. Na-K-ATPase and EGFR expressions were used as control. Quantification of the Western blots with respect to Na-K-ATPase are as shown for IL-20R1 (**B**), IL-22R1 (**C**), and IL-20R2 (**D**). DU-145 cells were treated with His-tagged MDA-7/IL-24 protein for the times indicated. The cells were stained with primary antibodies followed by Alexa fluor-488 tagged secondary antibodies and flow cytometry was done to study IL-20R1 (**E**), IL-22R1 (**F**), and IL-20R2 (**G**) expression. Statistical representation with respect to untreated control is shown by asterisks. ^*^ *P* < 0.05, ^**^ *P* < 0.01, ^***^ *P* < 0.001.

In the initial series of experiments, we sought to investigate whether MDA-7/IL-24 signaling involves internalization of the ligand/receptor complex. DU-145 cells were treated with His-tagged recombinant MDA-7/IL-24 and the abundancy of different subunits of the MDA-7/IL-24 receptor on the plasma membrane was examined by surface biotinylation and Western blotting. MDA-7/IL-24 treatment induced rapid (starting at 30 min of incubation) decrease in surface expression of the IL-20R1/IL-20R2/IL-22R1 receptor that indicates receptor internalization (Figure 1). Importantly cell surface expression of other abundant transmembrane proteins, Na-K ATPase and epidermal growth factor receptor (EGFR), was not changed upon MDA-7/IL-24 stimulation (Figure 1A). Quantification of the Western blotting was shown in Figure 1B–1D. As an alternative approach to measure ligand-dependent receptor internalization, we also used a flow cytometry analysis. DU-145 cells were treated with His-MDA-7/IL-24 at room temperature for different times (30–180 min). Fixed but not-permeabilized cells were incubated with different receptor subunit primary antibodies followed by Alexa-fluor-488 tagged secondary antibodies to selectively label receptors on the cell surface. Labeling intensity of the cells was measured by flow cytometry. In good agreement with the described surface biotinylation data, antibody labeling indicated a time dependent decrease of all receptor subunits in MDA-7/IL-24-stimulated DU-145 cells (Figure 1E–1G).

Finally, we sought to confirm MDA-7/IL-24-induced receptor internalization by using fluorescence microscopy. DU-145 cells were treated for 1 h with MDA-7/IL-24 either at conditions that blocked endocytosis (4° C), or at endocytosis-permissive conditions (room temperature) and endogenous receptor expression was analyzed. Cellular localization of IL-20R1, IL-20R2 and IL-22R1 receptor subunits was examined in fixed cells by immunolabeling and confocal microscopy. Under conditions of blocked endocytosis, all receptor subunits displayed similar localization at the cell cortex reflecting their enrichment at the plasma membrane (Supplementary Figure 1). By contrast, in endocytosis-permissive conditions, MDA-7/IL-24 stimulation caused redistribution of the receptor subunits from the cortex into different intracellular compartments including the perinuclear region (Supplementary Figure 1).

### Receptors undergo degradation upon internalization

Previous studies have demonstrated that upon activation with their ligands, several cytokine receptors became degraded via different mechanisms. For example, after internalization of the IL-2-IL-2R complex, IL-2 undergoes both lysosomal and proteosomal degradation [28, 29]. Furthermore, the IL-10 receptor undergoes proteosomal degradation upon activation and internalization [30]. Based on this data we decided to elucidate the fate of internalized IL-20/IL-22 receptors in MDA-7/IL-24-stimulated cancer cells.

**Figure 2:**
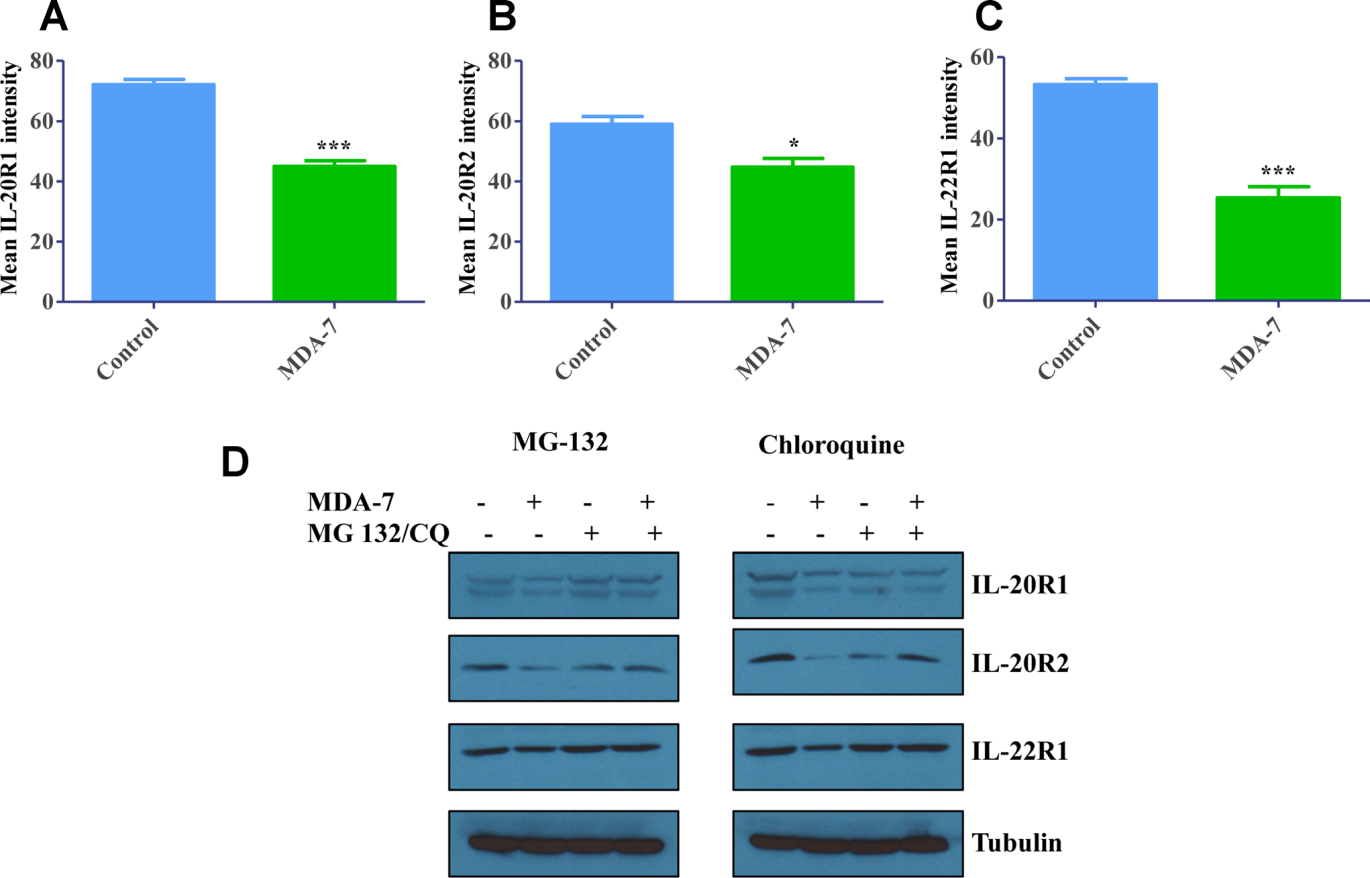
Degradation of Receptors upon internalization. (**A**) DU-145 cells were treated with His-MDA-7/IL-24 (10 μg/mL) for 24 hr. Flow cytometry analysis revealed that there was significant decrease in the total IL-20R1 after treatment with His-MDA-7/IL-24. Also, decrease in IL-20R2 (**B**) and IL-22R1 (**C**) expression was observed. ^*^ *P* < 0.05, ^***^ *P* < 0.001. (**D)** DU-145 cells were treated with His-MDA-7/IL-24 (10 μg/mL) plus MG-132 (5 μM) or chloroquine (10 nM) for 24 hr. Western blotting analysis of the receptors showed a decrease in IL-20R1/IL-20R2/IL-22R1 expression in DU-145 cells treated with His-MDA-7/IL-24 as compared to the untreated cells. In MG-132- or chloroquine-treated cells the decrease in expression was reversed.

DU-145 cells were treated with recombinant MDA-7/IL-24 protein and total cellular proteins were analyzed for receptor expression with flow cytometry. Significant decreases were observed in total receptor expression with MDA-7/IL-24 treatment showing degradation of receptors upon MDA-7/IL-24 treatment (Figure 2A–2C).

In order to identify the receptor degradation pathway, we used pharmacological inhibitors of either proteosomal (MG-132), or lysosomal (chloroquine) pathways. DU-145 cells were treated with MDA-7/IL-24 for 24 h, in combination with either MG-132, chloroquine (lysosomal inhibitor) or vehicle. Expression of different receptor subunits in total cell lysates was analyzed by Western blotting after 24 h of MDA-7/IL-24 exposure. Figure 2D shows that MDA-7/IL-24 exposure significantly decreased expression of IL-20R1, IL-20R2 and IL-22R1, which was reversed with either MG-132 or chloroquine treatment (Figure 2D and Supplementary Figure 2). These data suggest that internalized MDA-7/IL-24 receptor complexes are degraded through both proteosomal and lysosomal degradation pathways.

To examine intracellular behavior of exogenous IL-20R2 or IL-22R1 receptor subunits, we expressed these receptors in A549 lung adenocarcinoma cells that are known to express only IL-20R1 receptors and lack IL-20R2/IL-22R1 receptors [12]. This model permits testing internalization of different types of MDA-7/IL-24 receptors. A549 cells were transfected with vectors expressing either IL-20R2 or IL-22R1 receptors and treated with recombinant MDA-7/IL-24 protein. The abundancy of IL-20R1 receptors on the cell surface was examined by flow cytometry as described for DU-145 cells. Control A549 cells with only IL-20R1 receptors did not show any significant change in the surface expression of IL-20R1 receptors following treatment with MDA-7/IL-24 (Supplementary Figure 3A). By contrast, stimulation with MDA-7/IL-24 significantly decreased cell surface expression of IL-20R1 in those cells that expressed either exogenous IL-20R2, or IL-22R1 (Supplementary Figure 3B, 3C). Given the results of pharmacological inhibition, implicating lysosomes in degradation of MDA-7/IL-24 receptors, we sought to investigate if the receptor/ligand complexes are delivered to lysosomes. HeLa cells transfected with either CFP-IL-20R2, YFP-IL-20R1, or CFP-IL-22R1 were stimulated with MDA-7/IL-24 for 24 h and colocalization of the receptors with lysosomal markers, lysotracker red and LAMP1 were examined by fluorescence microscopy (Supplementary Figure 4A, 4B). Upon stimulation with His-MDA-7/IL-24, a significant accumulation of all CFP and YFP-tagged receptors in lysotracker and LAMP1-positive lysosomes was observed (Supplementary Figure 4A, 4B). These results reinforce our conclusion that MDA-7/IL-24 engagement directs its receptors for lysosomal degradation. Collectively, these data suggest that MDA-7/IL-24 triggers internalization of its functional receptors composed of IL-20R1/IL-20R2 or IL-20R1/IL-22R1 heterodimers.

**Figure 3:**
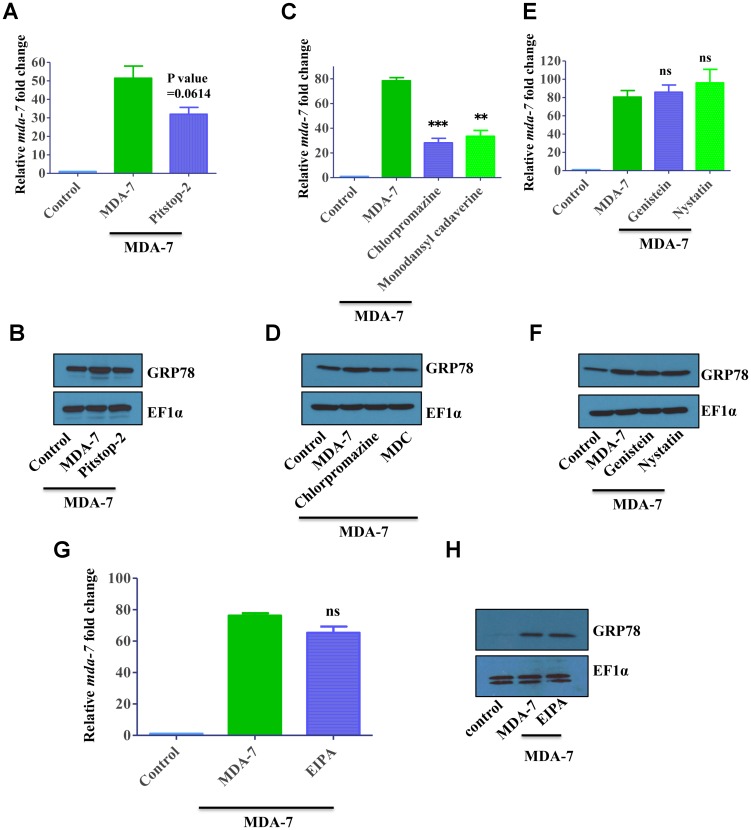
MDA-7/IL-24 uses the clathrin-mediated endocytic (*CME*) pathway. (**A**) DU-145 cells were treated with His-MDA-7/IL-24 (10 μg/mL) for 30 min with/without Pitstop-2. After 30 min fresh growth medium was added. Real time PCR was done to check the expression of *mda-7/IL-24*. (**B**) GRP78 expression was monitored by Western blotting analysis. MDA-7/IL-24 upregulated GRP78 expression, while in the presence of inhibitors, it failed to upregulate GRP78. (**C**) DU-145 cells were treated with His-MDA-7/IL-24 (10 μg/mL) for 30 min with/without chlorpromazine (10 μg/mL) or monodansylcadaverine (200 μM). After 30 min cells received fresh growth medium. Real time PCR was done to check the expression of *mda-7/IL-24*. (**D**) GRP78 expression was determined by Western blotting analysis. (**E**) DU-145 cells were treated with His-MDA-7/IL-24 (10 μg/mL) for 30 min with/without Genistein (200 μM) or Nystatin (20 μM). After 30 minutes fresh growth medium was added. Real time PCR was done to check the expression of *mda-7/IL-24*. (**F**) GRP78 expression was determined by Western blotting analysis. Genistein and Nystatin had no effect on MDA-7/IL-24-mediated activation of GRP78. (**G**) DU-145 cells were treated with His-MDA-7/IL-24 (10 μg/mL) for 30 min with/without EIPA (1 mM). After 30 minutes fresh growth medium was added. Real time PCR was done to check the expression of *mda-7/IL-24*. (**H**) GRP78 expression was determined by Western blotting analysis. EIPA had no effect on MDA-7/IL-24-mediated activation of GRP78. ^**^ *P* < 0.01, ^***^ *P* < 0.001, ns: not significant.

### MDA-7/IL-24 receptor complexes are internalized via clathrin-mediated, dynamin-dependent endocytosis

Next we sought to dissect the endocytic pathway that mediates internalization of MDA7/IL-24-receptor complexes in cancer cells. Several endocytic pathways have been identified, among them clathrin-dependent endocytosis, caveolin/lipid raft-mediated endocytosis and macropinocytosis are the best characterized [31]. In the initial series of experiments, we used pharmacological inhibitors that are relatively specific for major endocytic pathways. Chlorpromazine and monodansylcadaverine (MDC) are known to inhibit the clathrin pathway, genistein and nystatin are inhibitor of caveolin/lipid raft-dependent internalization, whereas ethyl-isopropyl amiloride (EIPA) blocks macropinocytosis [32].

We sought to investigate whether internalization of MDA-7/IL-24-receptor complexes can modulate functional activity of this cytokine. Two different functional readouts were used for these experiments. One is based on our previous findings that signaling of exogenous MDA-7/IL-24 induces expression of endogenous MDA-7/IL-24 in an autocrine fashion [19]. Therefore, we measured *mda-7/IL-24* transcript level as a functional readout of MDA-7/IL-24-receptor activation. Furthermore, since MDA-7/IL-24 is known to induce expression of GRP78, we used immunoblotting to examine GRP78 expression in MDA-7/IL-24-treated cells. As predicted, MDA7/IL-24 treatment upregulated endogenous *mda-7/IL-24* transcript levels and increased expression of GRP78 proteins in DU-145 cells. Cell activation with MDA-7/IL-24 in the presence of clathrin inhibitors Pitstop-2 or chlorpromazine or MDC significantly attenuated the increase in endogenous *mda-7/IL-24* and GRP78 expression (Figure 3A–3D, Supplementary Figure 5). By contrast, inhibitors of either caveolar-mediated endocytosis, or macropinocytosis did not attenuate MDA-7/IL-24-induced upregulation of endogenous *mda-7/IL-24* and GRP78 (Figure 3E–3H, Supplementary Figure 5). Collectively, these data suggest that clathrin-dependent internalization of MDA-7/IL-24 is required for the cytokine signaling from and yet to be defined intracellular compartment of cancer cells.

**Figure 4:**
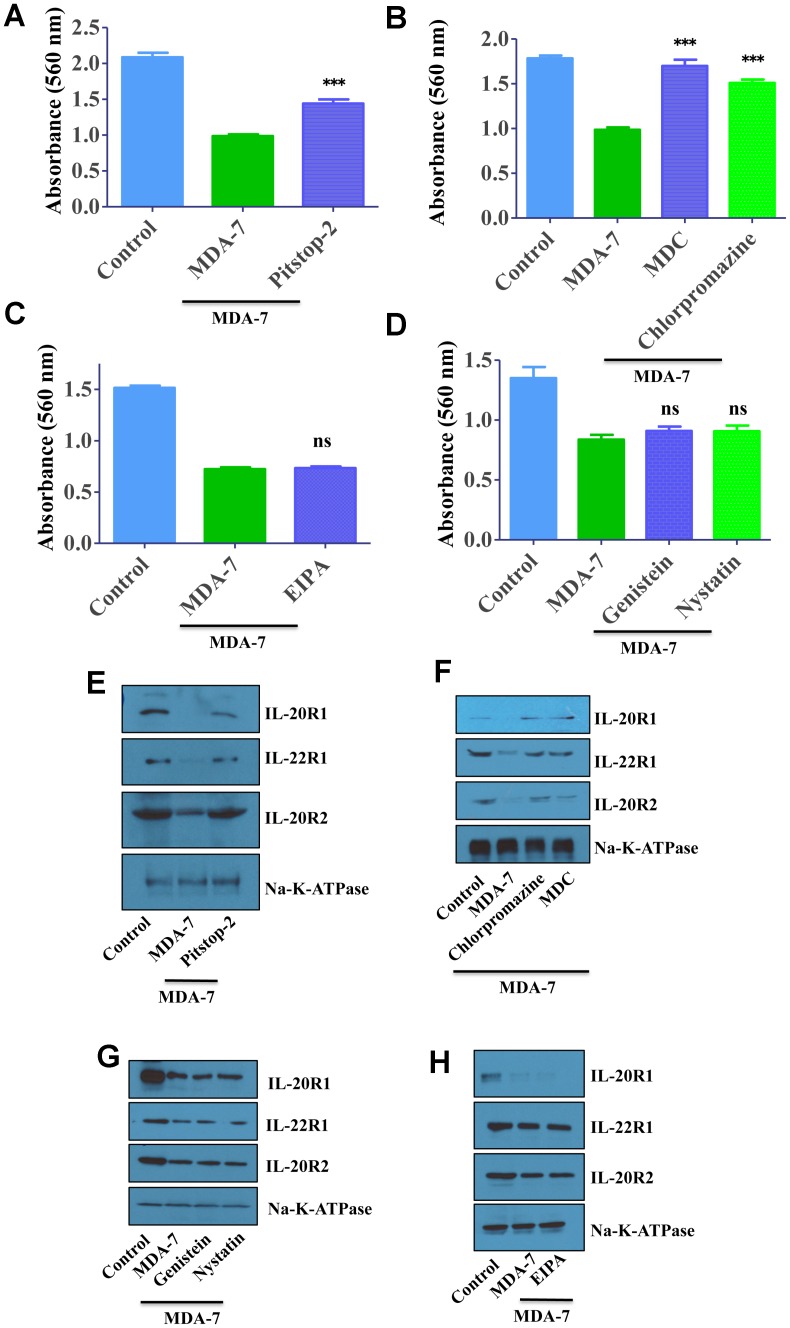
MDA-7/IL-24 uses the clathrin-mediated endocytic (*CME*) pathway. (**A**) DU-145 cells were treated with His-MDA-7/IL-24 (10 μg/mL) for 30 min with/without Pitstop-2. After 30 min fresh growth medium was added. MTT assay was done to study the effect of MDA-7/IL-24. MDA-7/IL-24 inhibited the proliferation of cells, while in the presence of inhibitors; it failed to inhibit the proliferation of cells. (**B**) DU-145 cells were treated with His-MDA-7/IL-24 (10 μg/mL) for 30 min with/without chlorpromazine (10 μg/mL) or monodansylcadaverine (200 μM). After 30 min cells received fresh growth medium. MTT assay was done to study the effect of MDA-7/IL-24. (**C**) DU-145 cells were treated with His-MDA-7/IL-24 (10 μg/mL) for 30 min with/without EIPA. After 30 min fresh growth medium was added. MTT assay was done to study the effect of MDA-7/IL-24. (**D**) DU-145 cells were treated with His-MDA-7/IL-24 (10 μg/mL) for 30 min with/without Genistein (200 μM) or Nystatin (20 μM). After 30 minutes fresh growth medium was added. MTT assay was done to study the effect of MDA-7/IL-24. ^***^ P<0.001, ns: not significant. (**E**) DU-145 cells were treated with His-MDA-7/IL-24 (10 μg/mL) for 30 min with/without Pitstop-2. Western blotting showing the expression of receptors, biotinylated membrane fraction was isolated and probed with the indicated antibodies. Na-K-ATPase was used as control. (**F**) DU-145 cells were treated with His-MDA-7/IL-24 (10 μg/mL) for 30 min with/without chlorpromazine (10 μg/mL) or monodansylcadaverine (200 μM). After 30 min cells received fresh growth medium. Western blotting showing the expression of receptors, biotinylated membrane fraction was isolated and probed with the indicated antibodies. (**G**) DU-145 cells were treated with His-MDA-7/IL-24 (10 μg/mL) for 30 min with/without Genistein (200 μM) or Nystatin (20 μM). Western blotting showing the expression of receptors, biotinylated membrane fraction was isolated and probed with the indicated antibodies. (**H**) DU-145 cells were treated with His-MDA-7/IL-24 (10 μg/mL) for 30 min with/without EIPA. After 30 min fresh growth medium was added. Western blotting showing the expression of receptors, biotinylated membrane fraction was isolated and probed with the indicated antibodies.

We performed MTT assay to validate the clathrin-mediated endocytic pathway of MDA-7/IL-24. As reported earlier, MDA-7/IL-24 inhibited the proliferation of DU-145 cells, whereas treatment of clathrin-inhibitors altered the inhibitory activity of MDA-7/IL-24 (Figure 4A, 4B). Treatment with caveolin inhibitors or macropinocytosis inhibitor did not alter the proliferation inhibition of MDA-7/IL-24. These results again confirm the clathrin-mediated endocytosis of MDA-7/IL-24 (Figure 4C, 4D).

To validate further these studies, membrane fractions were analyzed to check the clathrin-mediated endocytic pathway of MDA-7/IL-24. As reported earlier in MDA-7/IL-24-treated cells membrane receptor expression goes down, which is altered following treatment with clathrin-inhibitors (Figure 4E, 4F). Treatment with caveolin inhibitors or macropinocytosis inhibitor did not alter this phenomenon. These results again confirm the clathrin-mediated endocytosis of MDA-7/IL-24 (Figure 4G, 4H).

**Figure 5:**
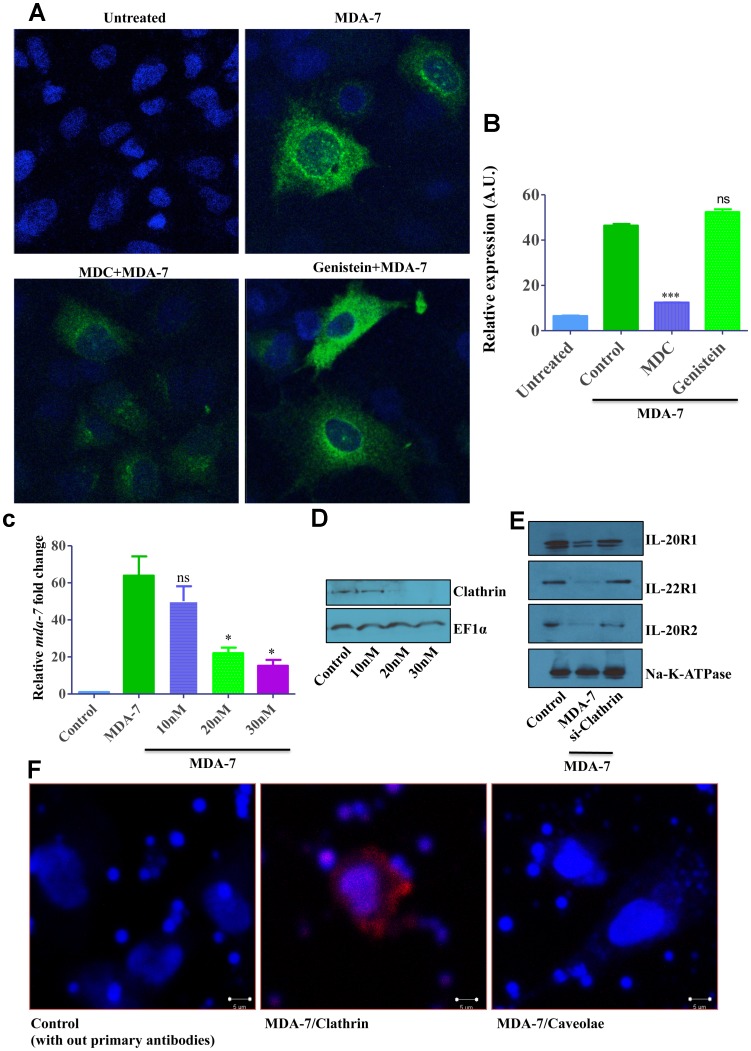
Fluorescent microscopy and Clathrin knockdown data showing clathrin-mediated endocytosis (CME) of MDA-7/IL-24. (**A**) DU-145 cells were treated with His-MDA-7/IL-24 (10 μg/mL) for 30 min with or without monodansylcadaverine (200 μM) or Genistein (200 μM). After 30 min cells were fixed, stained with MDA-7/IL-24 antibody and observed with confocal microscopy. Image quantification is as shown (**B**). ^***^ *P* < 0.001, ns: not significant. (**C**) DU-145 cells were transfected with different doses of clathrin siRNA, after 24 hr cells were treated with His-MDA-7/IL-24 (10 μg/mL). Real time PCR was done to check the expression of *mda-7/IL-24*. ^*^ *P* < 0.05, ns: not significant. (**D**) Knockdown of clathrin was shown by Western blotting. EF1α used as a loading control. (**E**) Western blotting showing the expression of receptors, biotinylated membrane fraction was isolated and probed with the indicated antibodies. (**F**) Proximity ligation assay was performed on DU-145 cells with the antibodies as shown.

To scrutinize this process further, internalization of exogenously added MDA-7/IL-24 was monitored in DU-145 cells exposed to different endocytosis inhibitors. The amount of internalized protein was determined by confocal microscopy using anti-MDA-7/IL-24 antibody after acid wash that removed non-internalized surface bound ligand. Cell activation with MDA-7/IL-24 resulted in cytokine internalization and accumulation in the perinuclear intracellular compartment (Figure 5A). This process was significantly attenuated in monodansylcadaverine (MDC)-treated cells, but was unaffected in the cells treated with caveolar-dependent endocytosis inhibitor (genistein) (Figure 5A, 5B). Together these pharmacological inhibition data suggest that activated MDA-7/IL-24-receptor complexes are internalized via the clathrin-mediated pathway.

Since all pharmacological inhibitors do not possess absolute specificity to the targeted endocytic pathways, we sought to confirm the results of our pharmacological analysis by using more specific genetic tools. First, RNA interference was used to downregulate expression of clathrin heavy chain (CHC). DU-145 cells were transfected with different concentrations of CHC siRNA and examined for the efficiency of CHC knockdown and the effects of such knockdown on MDA-7/IL-24 internalization and signaling. Western blotting analysis confirmed that 20–30 nM of CHC siRNA effectively downregulated this protein expression (Figure 5D). Such CHC downregulation significantly attenuated endocytosis of MDA-7/IL-24 (or cognate receptors) and markedly inhibited expression of endogenous *mda-7/IL-24* in stimulated cells (Figure 5C). Analysis of the membrane receptor expression showed a similar phenomenon (Figure 5E).

Additionally, we sought to investigate if MDA-7/IL-24 colocalizes with CHC in cancer cells by using a proximity ligation assay. DU-145 cells were treated with His-MDA-7/IL-24 and after 30 min of incubation they were fixed and processed as per the protocol suggested by the manufacturer for performing the proximity ligation assay. A positive colocalization signal was observed for the MDA-7/IL-24*-*CHC antibody pairs, but not for the MDA-7/IL-24-caveolin-1 antibody pair (Figure 5F), thereby indicating a specific association of internalizing MDA-7/IL-24 with CHC in nonfractionated cancer cells.

**Figure 6:**
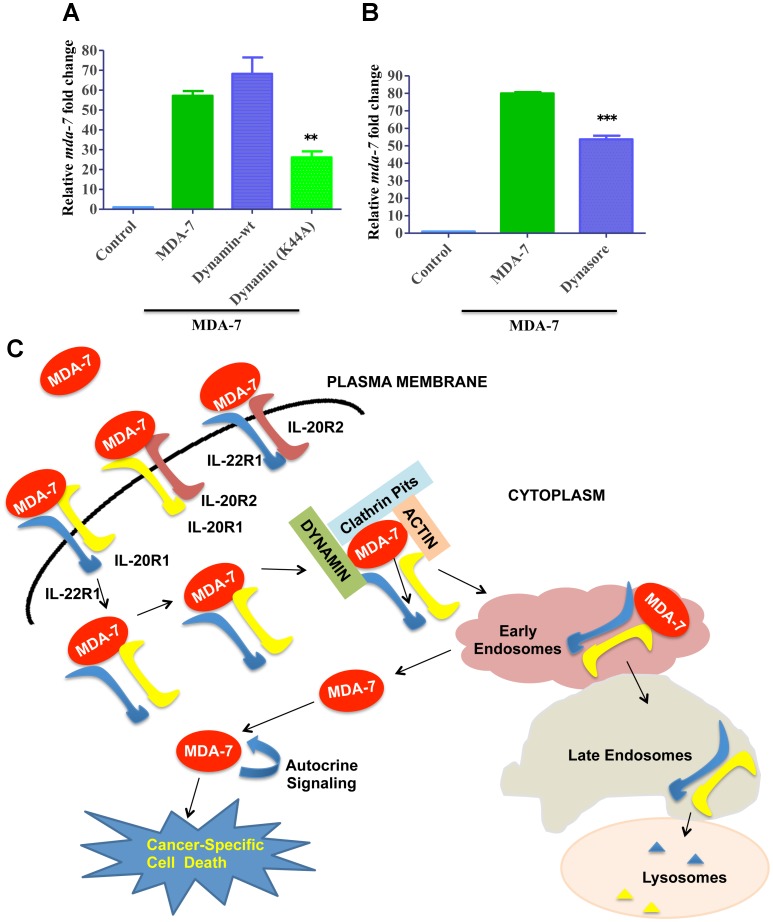
Role of Dynamin in MDA-7/IL-24 endocytosis. (**A**) DU-145 cells were transfected with Dynamin-wt or Dynamin-K44A mutant and subsequently treated with His-MDA-7/IL-24 (10 μg/mL). Real time PCR was done to check the expression of *mda-7/IL-24*. (**B**) DU-145 cells were treated with His-MDA-7/IL-24 (10 μg/mL) for 30 min with/without Dynasore. After 30 min fresh growth medium was added. Real time PCR was done to check the expression of *mda-7/IL-24*. ^**^ P<0.01, ^***^ *P* < 0.001. (**C**) Schematic representation of MDA-7/IL-24 endocytosis: MDA-7/IL-24 internalizes into the cell by a receptor-dependent mechanism. The cytokine undergoes clathrin-mediated endocytosis, in which dynamins play important roles. The receptors undergo degradation and MDA-7/IL-24 is released into the cell to induce downstream signaling cascades.

Dynamin is a large GTPase, which is involved in different endocytic processes by mediating separation of endocytic vesicles from the plasma membrane [33]. Since formation of clathrin-coated vesicles is known to be dependent on dynamin [33], we sought to investigate if dynamin is involved in MDA-7/IL-24 endocytosis by using both genetic and pharmacological approaches. The genetic approach involved overexpression of a dominant-mutant of dynamin (K44A Dynamin) lacking GTP binding, which has been widely used to probe dynamin function in different cells [33]. The pharmacological approach involved a chemical inhibitor of dynamin, Dynasore [33]. Figure 6A shows that overexpression of dominant-mutant dynamin significantly attenuated internalization of MDA-7/IL-24 in DU-145 cells, whereas overexpression of wild-type dynamin did not significantly affect this process. Similar inhibition of MDA-7/IL-24 internalization was observed in dynasore-treated cells (Figure 6B). A schematic representation of the results of this study is shown in Figure 6C.

## DISCUSSION

*mda-7/IL-24* has been validated as a potent therapeutic for multiple cancers *in vitro* and *in vivo* [1, 2, 6, 34–36]. Transduction of tumor or normal cells with the *mda-7/IL-24* gene results in cancer-specific cell death [3, 7, 10]. Secreted MDA-7/IL-24 protein functions as a pro-Th1 cytokine and as a potent antiangiogenic molecule [37]. The pure cytokine pairs with the surface IL-20/IL-22 heterodimeric receptor complex to internalize into cells [38]. Recently the crystal structure of IL-24 with its dimeric receptors, IL-22R1 and IL-20R2 has been established [38]. The study also suggests that IL-20R2 might be the high affinity receptor [38]. This MDA-7/IL-24 receptor engagement results in up regulation of BAX protein leading to apoptosis induction [3]. Also, MDA-7/IL-24 upon internalization in cells interacts and up regulates the chaperone protein BiP/GRP78 to induce cancer-specific cell death without harming normal cells [16]. Using the prostate cancer cell line DU-145, which has a complete set of cognate receptors, we demonstrate that MDA-7/IL-24 interacts with these receptors leading to protein and receptor internalization. Time point kinetics data show that at 1hr incubation with the ligand protein, there is significant receptor internalization.

An MDA-7/IL-24 autocrine/paracrine loop up regulates its own transcript level [19]. This finding helps explain the potent “bystander” antitumor effects of this secreted cytokine, seen *in vivo* in animal models and in a Phase I/II clinical trial in patients with advanced cancers [1, 2, 4–6, 34, 39, 40]. In a phase I/II clinical trial a replication incompetent adenovirus expressing *mda-7/IL-24,* Ad.*mda-7* (INGN 241) (2 × 10^10^ to 2 × 10^12^ vp) *was administered* intratumorally in a cohort of 28 patients with melanomas and advanced carcinomas and injections were repeated in a dose-escalating manner. All the treated lesions showed vector transduction, vector specific DNA and RNA expression, and an elevated MDA-7/IL-24 protein. Vector DNA and mRNA were detected in nearby areas of the injection site with high intensity, whereas MDA-7/IL-24 protein could be detected many centimeters away from the injected site. Apoptosis induction was also evident near the injection site and in distant lesions. In the treated group 44% clinical response was achieved in injected lesions. The clinical trial revealed that intratumoral administration of MDA-7/IL-24 was safe and well-tolerated, and has promising therapeutic activity [40].

We used this autocrine/paracrine loop to scrutinize the endocytic mechanism of cellular entry of MDA-7/IL-24. There are different endocytic pathways, i.e., receptor-mediated endocytosis and receptor-independent endocytosis. We used the macropinocytosis inhibitor EIPA and showed that MDA-7/IL-24 entry into cells does not proceed through a receptor-independent endocytosis pathway. Moreover, A549 cells, which lack a functional pair of canonical IL-20/IL-22 receptors, do not respond to recombinant MDA-7/IL-24 protein [12]. The autocrine up regulation was inhibited by the use of inhibitors of clathrin-mediated endocytosis, but not by inhibitors of caveolae-mediated endocytosis.

Receptor-mediated endocytosis at the plasma membrane is initiated by several internalization mechanisms. The traditional pathway involves clathrin protein or clathrin-mediated endocytosis (or *CME*) and clathrin-independent endocytosis (or *CIE*). We document that MDA-7/IL-24 undergoes *CME* and the receptors undergo both lysosomal and proteosomal degradation after they enter late endosomes. This is similar to IL-10R1, which undergoes ligand-dependent internalization leading to proteosomal degradation [30].

A number of surface receptors are ubiquitinated upon ligand activation, some examples include platelet derived growth factor receptor [41], TCR [42], c-kit [43], and growth hormone receptor [44–46]. They are ubiquitinated following ligand activation with lysosomes serving as primary degradation effectors. Previous studies by Gopalan *et al*. [47] indicated that MDA-7/IL-24 was ubiquitinated and regulated by the ubiquitin-proteasome system, with inhibition of MDA-7/IL-24 degradation enhancing antitumor activity in ovarian and lung cancer cells. The present studies (Figure 2D and Supplementary Figure 2), suggest that IL-20R1, IL-20R2 and IL-22R1 receptors are degraded through the ubiquitin-proteosomal degradation pathway (MG-132 treatment) and lysosomal degradation pathway (chloroquine treatment) in the presence of MDA-7/IL-24.

The GTPase protein dynamin is essential for *CME* [48]. Dynamins function crucially in cellular signaling, nutrient uptake, and in maintenance of cellular homeostasis [48]. Dynamin’s GTPase activity and GTP-dependent conformational changes result in self-assembly into a collar around the neck of the coated pits or the invagination. To delineate the role of dynamin in MDA-7/IL-24 internalization, we transfected dynamin and its GTPase mutant K44A into cells treated with MDA-7/IL-24. Interestingly, we observed that in Dynamin K44A transfected cells, MDA-7/IL-24 failed to up regulate *mda-7/IL-24* transcripts as compared to wild type dynamin transfected cells. This was also confirmed by the use of a chemical inhibitor of dynamin, Dynasore. The dynamin actin cytoskeleton and its polymerization also play important roles in *CME* [26]. Dynamin activity was shown to regulate different processes during *CME*, i.e., formation of clathrin pits, invagination/constriction, and scission of clathrin vesicles [26].

Using pharmacological and genetic approaches, we have now deciphered the mechanism of MDA-7/IL-24 internalization and endocytosis. We confirm that internalization occurs following ligand interaction with its cognate receptors and both the protein and the receptor are internalized. The role of clathrin and *mda-7/IL-24* in regulating cell surface receptor degradation can lead to various new downstream pathways, that are different in normal vs. cancer cells, and new potential cancer drug targets maybe identified by exploiting these pathways.

## MATERIALS AND METHODS

### Plasmids, and cell cultures

Cell lines used in this study were DU-145 (human prostate cancer cell line), A549 (human Lung cancer cell line), RWPE-1, HEK-293, and HeLa (cervical cancer cell line), which were obtained from the ATCC (American Type Culture Collection) (Manassas, VA, USA). The IM-PHFA cell line was described previously [49]. All cell lines were routinely checked for mycoplasma contamination using commercial kits. All of these cell lines were purchased recently (within the last 3-years) and were strictly maintained as recommended by the manufacturer. Dynamin and Dynamin K44A plasmids were obtained from Addgene, (Cambridge, MA, USA). CFP and YFP vectors were purchased from Clontech (Mountain View, CA, USA). CFP and YFP tagged receptors were cloned using standard cloning procedures [50].

### Purification of MDA-7/IL-24 recombinant protein

Recombinant His-MDA-7/IL-24 was produced and purified as described previously [12, 19].

### Reagents and inhibitors

Pitstop 2 was purchased from Abcam (Cambridge, MA, USA). MDC (monodansyl cadaverine), MG-132, Chloroquine, Chlorpromazine, Genistein, Nystatin, EIPA, and Dynasore were from Sigma-Aldrich (St. Louis, MO, USA). Lysotracker Red was from Invitrogen, Thermo Fisher Scientific (Waltham, MA). Clathrin siRNA was from Santa Cruz Biotechnology (Dallas, TX, USA).

### Immunolabelling and confocal microscopy

Cells were cultured on coated chambered slides. The cells were treated/transfected as described in figure, washed in PBS, fixed with 4% paraformaldehyde (Sigma, USA) and permeabilized with 1:1 mixture of methanol: acetone. Cells were stained with primary antibodies for overnight and Alexa fluor tagged secondary antibodies (Invitrogen, USA) for 40 mins. Cells were mounted with a mounting medium containing DAPI. The slides were observed in laser scanning confocal microscope (Zeiss LSM 710, Germany).

### Flow cytometry based protein expression

For membrane receptor expression, cells were washed, stained with primary followed by Alexa-Fluor tagged secondary antibody. For total receptor expression cells were permeabilized and then stained as described above. Fluorescence signals were measured with BD FACS Canto flow cytometer (BD Biosciences, San Jose, CA). Data were analyzed with FACs DIVA software (BD Biosciences).

### Surface biotinylation and membrane protein isolation

Membrane proteins were isolated using the Pierce Cell Surface Protein Isolation Kit (Thermo Fisher Scientific, USA) as per the manufacturer’s instructions. Briefly, cells were biotinylated (EZ-Link Sulfo-NHS-SS-Biotin) in ice-cold PBS, quenched (ice-cold PBS+ buffer plus 0.1% glycine), and lysed. The lysate was passed through the Neutr-Avidin agarose columns. The columns were washed and bound proteins were eluted with the elution buffer.

### siRNA and real-time quantitative PCR

To knockdown the expression of Clathrin, specific Clathrin heavy chain siRNA and its negative control were transfected into DU-145 cells using the Lipofectamine transfection reagent (Invitrogen, USA). RQ-PCR was performed on cDNA prepared from total RNA isolated from cells with an RNA isolation kit from Qiagen (Valencia, CA, USA). The TaqMan probes and master mix were obtained from Applied Biosystems (Foster City, CA, USA). Graph pad prism software was used for analyzing data.

### Western blotting

Standard protocols were followed for Western blotting assays [12]. The primary antibodies used were LAMP1, Tubulin, Dynamin, Clathrin, Caveolae, Na-K ATPase (Cell Signaling Technology, Boston, MA, USA), IL-20R1, IL-20R2, IL-22R1 (Abcam), GRP78 (Santacruz Biotechnology), and MDA-7/IL-24 (Genhunter, Nashville, TN, USA). Secondary antibodies used in this study were from Cell Signaling Technology.

### Proximity ligation assay

Proximity ligation assay (PLA) was performed with Duolink PLA kit from Sigma-Aldrich as described by the manufacturer. Briefly, cells were fixed, blocked with the blocking reagent and incubated with primary antibodies (anti-MDA-7/IL-24 and anti-clathrin or anti-caveolae antibodies) overnight at 4 °C. This was followed by incubation with oligonucleotides (anti-mouse and anti-rabbit PLA probes) conjugated secondary antibodies. Ligation and amplification steps were performed and PLA signals were detected under fluorescence microscope.

### Statistical analyses

Statistical analyses were performed by ANOVA or *t*-test using Graph pad prism software. *P*-value < 0.05 was considered significant.

## SUPPLEMENTARY MATERIALS


