# Supplementary material for: Mechanism of internalization of MDA-7/IL-24 protein and its cognate receptors following ligand-receptor docking
Source: Oncotarget. 2019 Aug 20;10(49):5103–17. doi: 10.18632/oncotarget.27150 (PMC6707942; doi:10.18632/oncotarget.27150)
Supplement: Supplementary file 1 [file oncotarget-10-5103-s001.pdf]

# Mechanism of internalization of MDA-7/IL-24 protein and its cognate receptors following ligand-receptor docking

## SUPPLEMENTARY MATERIALS

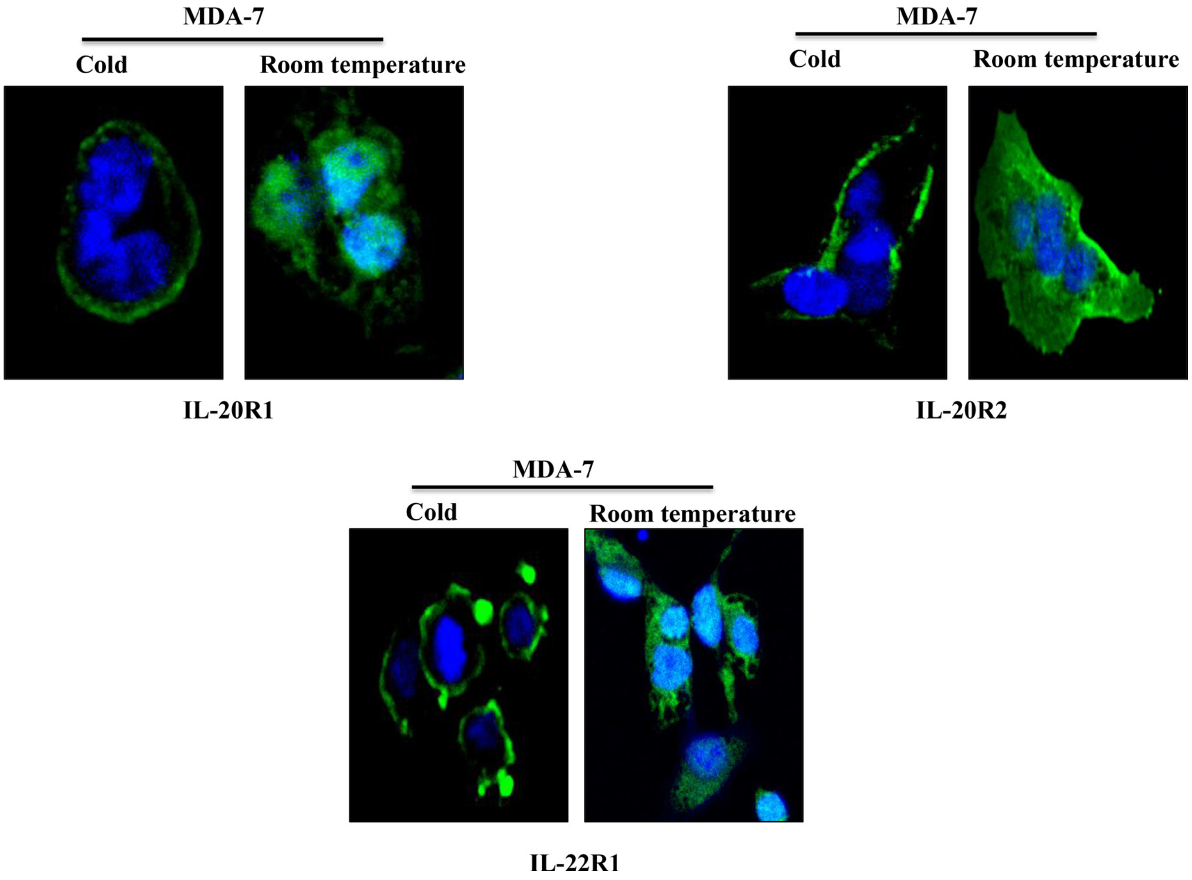


**Supplementary Figure 1: Internalization of receptors upon MDA-7/IL-24 treatment: DU-145 cells were treated with His-MDA-7/IL-24 (10 µg/mL) on ice or at room temperature for 1 hr.** Cells were then fixed and stained with respective antibodies followed by confocal microscopy.

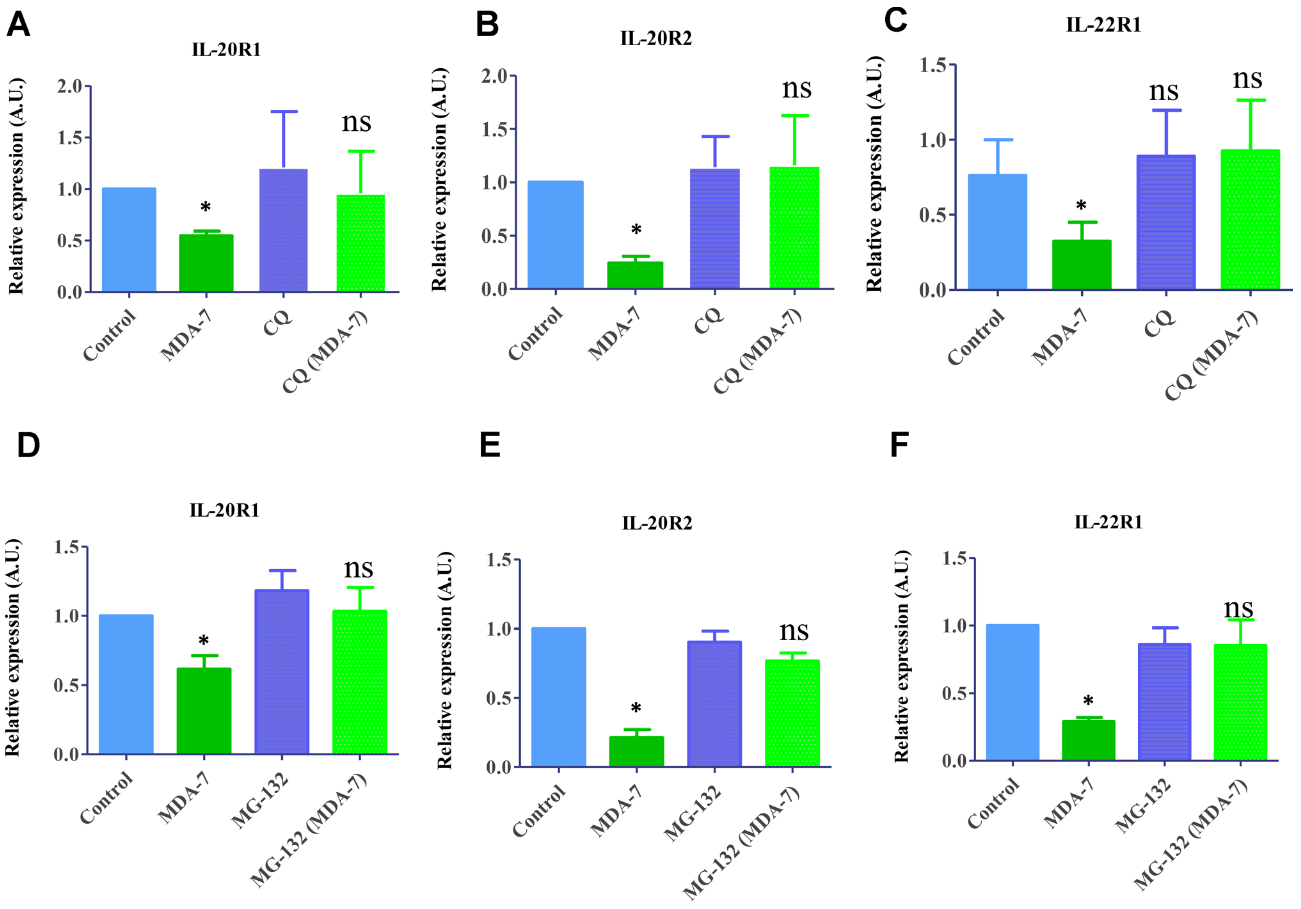


**Supplementary Figure 2: Quantification of the Western blots shown in Figure 2D.** Western blot images in Figure 2D were quantified using Image J software. $^{*}P < 0.05$, ns: not significant.

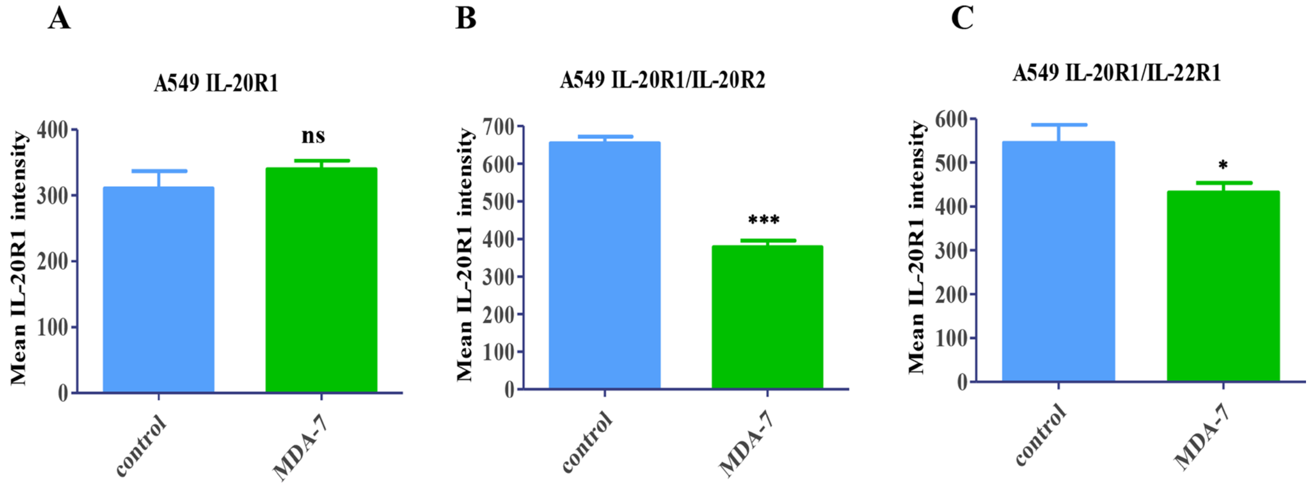


**Supplementary Figure 3: Degradation of receptors upon internalization.** A549 cells were transfected with vectors expressing either IL-20R2 or IL-22R1 and treated with His-MDA-7/IL-24 (10 μg/mL) for 24 hr. (A) Flow cytometry analysis revealed that there was no change in the total IL-20R1 after treatment with His-MDA-7/IL-24 in parental A549 cells without transfection. With transfection of IL-20R2 (**B**) and treatment with His-MDA-7/IL-24 (10 μg/mL) for 24 hr, a decrease in the total IL-20R1 expression was observed. (**C**) IL-22R1 was transfected and His-MDA-7/IL-24 (10 μg/mL) was added for 24 hr. Analysis of IL-20R1 expression showed that there was a decrease in the IL-20R1 expression. $^{*}P < 0.05$, $^{***}P < 0.001$, ns: not significant.

**A**

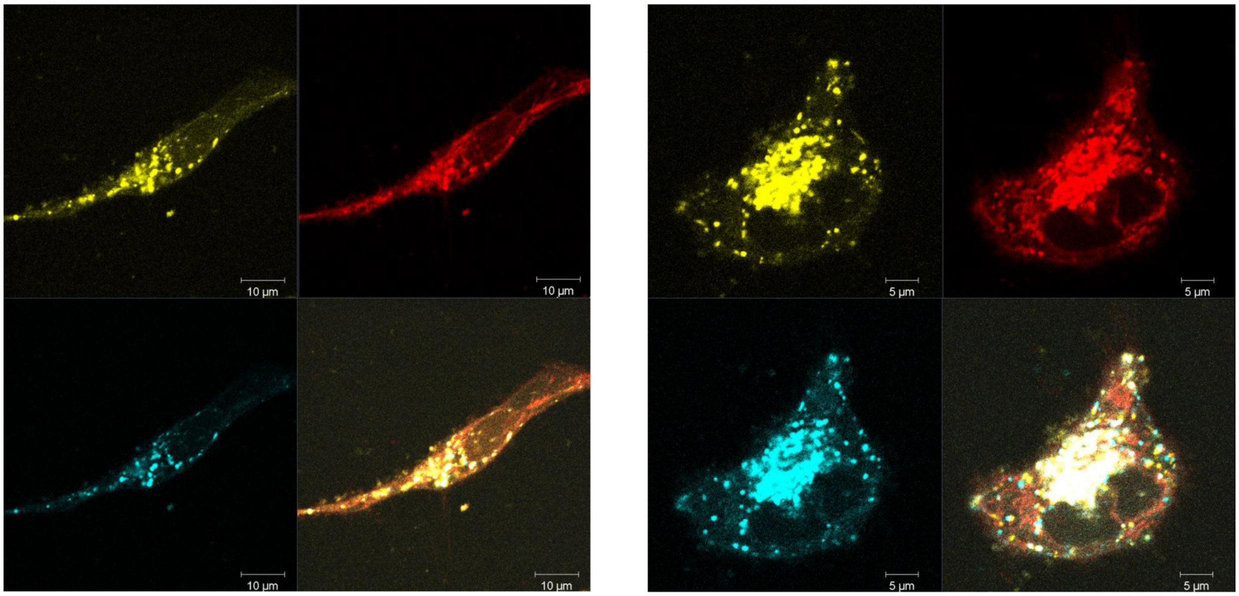


**B**

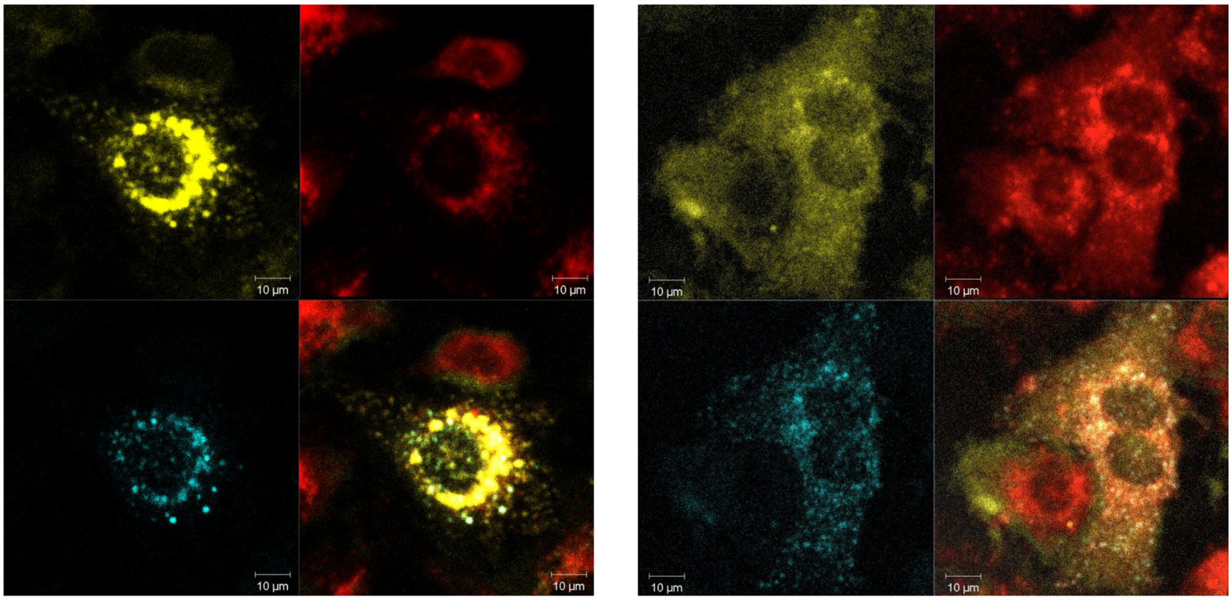


**Supplementary Figure 4: Colocalization of receptors with lysosomes.** (**A**) HeLa cells were transfected with YFP-IL-20R1 and CFP-IL-20R2 or CFP-IL-22R1 and treated with His-MDA-7/IL-24 (10 μg/mL) for 24 hr. Image analysis showed that the receptors colocalized with lysosomes, which stained with Lysotracker red. (**B**) LAMP1 staining was done to study the colocalization of the receptors with lysosomes.

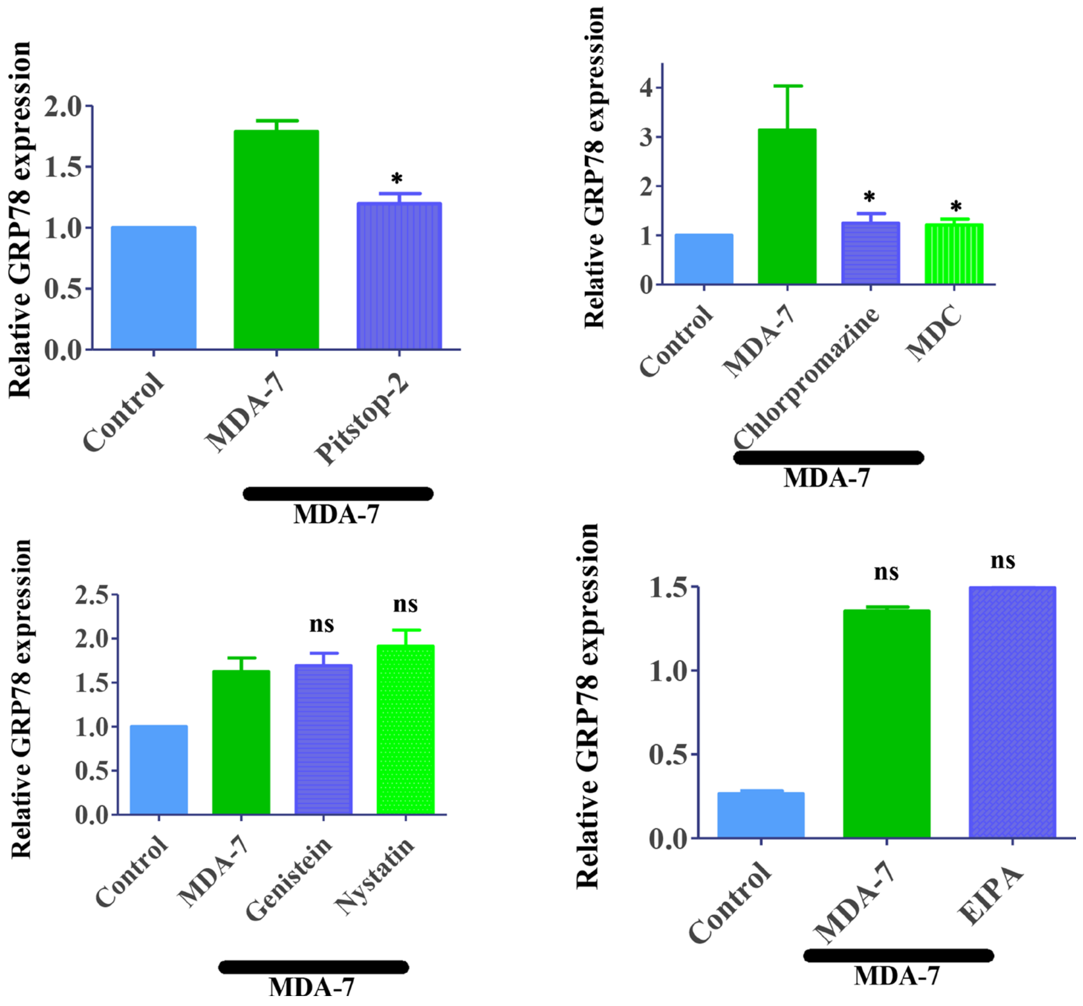


**Supplementary Figure 5: Quantification of the Western blots shown in Figure 3B, 3D, 3F, and 3H.** Western blot images in Figure 3B, 3D, 3F, and 3H were quantified using Image J software. $^{*}P < 0.05$, ns: not significant.
